# Kidney health among adolescents living in a high-risk region for CKDu

**DOI:** 10.1186/s12882-026-05070-1

**Published:** 2026-06-04

**Authors:** Selene Vences Brown, Yirong Yuan, Samantha M. Hall, Juan José Amador Velázquez, Damaris López Pilarte, Magaly Rosario Amador Amador Sánchez, Juan José Amador Sánchez, Josefa Elena Moya González, Alfonso Cesar Boza Gaitan, Stephanie Gonzalez Gil, Madeleine K. Scammell, David J. Friedman, Daniel R. Brooks, Jessica H. Leibler

**Affiliations:** 1 Department of Environmental Health, Boston University School of Public Health, Boston, MA 02118 USA; 2 Centro Médico del Pacífico (CENMED), Masaya, Nicaragua; 3 Division of Nephrology, Department of Medicine, Beth Israel Deaconess Medical Center, Harvard Medical School, Boston, MA USA; 4 Department of Epidemiology, Boston University School of Public Health, Boston, MA USA

**Keywords:** Mesoamerican nephropathy, Chronic kidney disease of uncertain etiology (CKDu), Heat exposure, Renal insufficiency, Central America

## Abstract

**Background:**

An epidemic of chronic kidney disease of uncertain origin (CKDu) imposes devastating mortality and morbidity in Central America, where the disease is termed Mesoamerican Nephropathy (MeN). MeN typically presents in young men working in heat-intensive manual jobs, notably agriculture, although the full disease etiology is unclear. Kidney health among young people in the region, particularly among females and those not employed in agriculture, is poorly characterized, limiting prevention efforts.

**Methods:**

We analyzed questionnaire and biospecimen data collected in 2023 from adolescents and young adults residing in five regions in Nicaragua, a country heavily impacted by the MeN epidemic (*n* = 568). Poor kidney health was defined as estimated glomerular filtration rate (eGFR) of < 90, <75, and < 60 mL/min/1.73 m², per international guidelines. We characterized an early MeN high-risk profile as eGFR < 75 mL/min/1.73 m² absent comorbid high BMI, diabetes, or hypertension. Employment, sociodemographic, and environmental predictors of low eGFR were assessed using inverse‑frequency weighted multivariable and bootstrapped LASSO‑penalized logistic regression.

**Results:**

Median eGFR was 106.7 mL/min/1.73 m² (IQR 16.3). The prevalence of eGFR < 90, <75, and < 60 mL/min/1.73 m² was 17.8% (*n* = 101), 4.1% (*n* = 23), and 1.1% (*n* = 6), respectively. EGFR < 75 mL/min/1.73 m² was more common in males than females (6.3% vs. 2.0%; *p* = 0.001). The MeN high‑risk profile was identified in 1.6% of participants (9/568), all males. Sugarcane employment in the last year was associated with higher odds of eGFR < 90 mL/min/1.73 m² (aOR 2.03; 1.10, 3.88; LASSO mOR 1.35; 1.00, 3.82). High BMI was associated with eGFR < 90 mL/min/1.73 m² (aOR 1.54; 1.16, 2.06; LASSO mOR 1.22; 1.00, 2.07). Other exposures, including residential drinking water source, low socioeconomic status, and non‑sugarcane heat‑intensive labor, were not significantly associated with low eGFR.

**Conclusions:**

We observed evidence of poor kidney health among Nicaraguan adolescents and young adults. A MeN high-risk profile was observed exclusively among young males and included individuals without occupational heat exposure. Our findings suggest elevated prevalence of multiple profiles of kidney dysfunction among young people in the region not fully explained by occupational exposures. Further surveillance of kidney health among young people may shed light on early life risk factors for this devastating disease.

**Supplementary Information:**

The online version contains supplementary material available at 10.1186/s12882-026-05070-1.

## Introduction

Chronic kidney disease of uncertain etiology (CKDu) is a complex and devastating disease with hotspots worldwide, including Central America, where the disease is termed Mesoamerican Nephropathy (MeN) [1–4]. MeN typically presents with minimal or no proteinuria, tubulointerstitial damage, small kidney size, and absence of metabolic comorbidities, such as diabetes, hypertension, and obesity, which are established risk factors for traditional CKD [5–7]. Occupational heat stress is an established risk factor for MeN, with some research indicating genetic predispositions for disease, although comprehensive disease etiology remains unclear [8–11]. Beyond individual health impacts, MeN imposes substantial social and economic burdens on affected communities, where the loss of young adult males can exacerbate household financial instability, strain already limited social support systems, reduce economic opportunity for youth, and contribute to intergenerational poverty [12]. 

Emerging evidence from Central America and other CKDu hotspots in Sri Lanka and India underscores the hypothesis that CKDu is a multifactorial disease process that begins in early life, with evidence of kidney health dysfunction visible during childhood and adolescence, prior to work experience [13]. Our research and that of others has identified biomarkers and molecular signatures of poor kidney health in Central America among non-working youth and young adults, including among females [14–18]. In occupational studies, the identification of extremely rapid kidney function decline among a small number of young male laborers suggest that occupational exposures later in life may interact with preexisting physiological or genetic susceptibility to result in rapid disease initiation or progression [18–21]. 

Additionally, variability in CKDu presentation across hotspot regions highlights the potential role of nonoccupational exposures, including drinking water quality, agrochemicals, infectious disease, and nutritional stressors [22–25]. These findings point to the importance of characterizing kidney health trajectories beginning early in life to clarify disease pathways and inform prevention strategies in high risk regions.

In 2023, we assessed cross-sectional kidney health within a community cohort of male and female adolescents and young adults in Nicaragua (the "Jovenes-Nica" Study), a country with CKDu mortality rates among the highest in the world [26, 27]. We describe the distribution of eGFR in this cohort and report prevalence and risk factors for low eGFR (< 90, < 75, and < 60 ml/min/1.73 m^2^). Additionally, we describe a high-risk MeN risk profile, consisting of eGFR < 75 ml/min/1.73 m^2^ without comorbid diabetes, hypertension, or high BMI, which are key risk factors for kidney disease in other regions [28]. Our objectives were to characterize population distribution of kidney function in adolescence and young adulthood and identify demographic and occupational risk factors for low eGFR in this high-burden setting.

## Methods

### Study design

This study includes participants recruited and enrolled in two waves, first in 2011 (*n* = 245; ages 12–19 years) and again in 2016 (*n* = 659; 7–12 years), in the municipalities of Mina el Limón, Masaya, Managua, Chichigalpa, La Paz Centro, and Jinotega in Nicaragua (*N* = 904). These communities were selected to include high-risk regions MeN (Chichigalpa, La Paz Centro, Mina el Limón) as well as moderate risk (Masaya) and low risk (Jinotega, Managua). Regions such as Masaya and Managua are peri-urban and urban regions, respectively, that are occupationally dominated by commerce and commercial textiles. Jinotega is the largest coffee production region in Nicaragua, Mina el Limón is dominated by the mining industry, and Chichigalpa is the largest sugarcane agricultural region in the country and the center of the MeN epidemic in Nicaragua [1–3].

With expanded funding, we followed up with these participants in 2022 after an extended period of no contact to enroll them in a new prospective study with three annual visits over three years (2022–2025). The team successfully re-engaged 71.1% (*n* = 643) of study participants using extensive field outreach [18]. Additional details on cohort design and follow up are provided elsewhere [14–18]. The current manuscript includes cross-sectional data collected in 2023 (*n* = 568). All study protocols were approved by the Institutional Review Board at Boston University Medical Center and the Nicaraguan Ministry of Health. The study was conducted in accordance with the ethical principles outlined in the Declaration of Taipei, including provisions related to informed consent and the responsible use of secondary health data and biological specimens.

### Interviews

Study participation included a structured interview with questions on employment, education, medical history, household exposures, pesticide use, diet, and behaviors. Interviews were conducted in person by a trained field team member using a tablet with the REDCap mobile app. All study visits were conducted in Spanish by Nicaraguan staff members trained in the study protocols. Field team members reviewed and provided feedback on the study protocols and questionnaires to ensure cultural competence and overall contextual understanding of questions. Biospecimen collection and laboratory protocols were provided in detailed written format and visually with pictographs.

### Biospecimen collection

Clean-catch urine and blood specimens were collected by professional phlebotomists following standard collection protocols. All specimens were maintained at -20 °C for less than 1 week, transferred to -80 °C at the Nicaraguan Ministry of Health (MINSA) National Laboratory in Managua, and shipped to the US for long-term storage at -80 °C until analysis. *Laboratory*: Serum specimens were analyzed at Quest Diagnostics in Boston, MA using the basic renal function panel (albumin, BUN/creatinine Ratio, calcium, carbon dioxide, chloride, creatinine, glucose, phosphorus, potassium, sodium, and urea nitrogen (BUN) (Supplemental materials).

### Primary outcome

*eGFR*: Estimated glomerular filtration rate (eGFR) was assessed using the age- and sex-adjusted serum creatinine-based European Kidney Function Consortium (EKFC) equation [29]. This equation has been used across population studies in international settings among assumed healthy populations to estimate eGFR and has particular application in modeling kidney health across the adolescent transition to adulthood [30]. We used the Kidney Disease Improving Global Outcomes (KDIGO) threshold of < 90 ml/min/1.73 m^2^ to characterize early reduction in kidney function (“low eGFR”) in this age group [31]. In addition, we estimated prevalence of eGFR < 75 and < 60 ml/min/1.73 m^2^. Specifically, we evaluated the eGFR < 75 ml/min/1.73 m^2^ cutoff as a evidence-based marker of poor kidney health in an adolescent and young adult population as an effort to provide a more nuanced understanding of early kidney health risk in this young population [29–31]. EGFR < 75 was used for descriptive and sensitivity analyses and to define a putative MeN high-risk profile (below), whereas eGFR < 90 was retained for primary comparability with prior analyses and for regression modeling.

### MeN high-risk profile

We also developed a putative MeN high-risk profile to differentiate between participants with early indications of MeN compared to individuals with indications of other CKD profile. For this analysis, the MeN high-risk profile was defined as eGFR < 75 ml/min/1.73 m^2^ and no self-reported history of diabetes, hypertension or high BMI from participant interviews, using established epidemiologic definitions of MeN [3, 5, 16, 21, 32]. As noted above, this eGFR cutoff was selected to enrich for more pronounced reductions in kidney function while remaining above the conventional eGFR < 60 ml/min/1.73 m^2^ threshold, for which counts were low; it is not intended to serve as a diagnostic definition of CKD in this epidemiological context.

### Statistical analysis

In regression models, we assessed predictors of low eGFR defined in primary analyses as eGFR < 90 mL/min/1.73 m². Due to low counts at lower eGFR thresholds, we were unable to engage in primarily regression modeling with these endpoints with precision, although we did explore eGFR < 75 mL/min/1.73 m² in sensitivity analyses described below. Model covariates were informed by the literature and included sex (male or female), age (years, continuous), residential drinking water source (municipal or other), BMI, and low socioeconomic status (SES) [1, 8, 13, 33, 34]. An SES variable was developed as the ratio of the number of income earning household members divided by the total household size in number of persons. Low SES was defined as the lower 50th percentile of this ratio [35]. 

We developed a mutually exclusive three‑level exposure variable to characterize heat‑intensive manual labor. Occupational experience and employment were derived from detailed questionnaire data based on in-person interviews. We used the literature and in-country expert engagement to characterize heat exposure [18]. A heat-intensive occupational variable included all workers who currently worked or reported primary or secondary employment in the last year in the following industries that were characterized as heat-intensive: sugarcane, basic grain farming, construction, herding, mining, or clay/brick/tilemaking (Table 2). In more detailed analyses, we separated sugarcane workers from others within this heat-intensive designation to distinguish specific impacts of sugarcane work, due to the relevance of this industry in MeN disease presentation.

### Regression analysis

To address outcome class imbalance (eGFR < 90 vs. ≥ 90), we fit a weighted multivariable logistic regression using inverse‑frequency (class) weighting, where observations with eGFR < 90 were weighted by n_0_/n_1_ and observations with eGFR ≥ 90 were weighted 1 [36]. This approach improves model stability and estimation in the presence of an imbalanced binary outcome, while confounding was addressed through multivariable adjustment. We fit a LASSO‑penalized logistic regression model (binomial family, α = 1) to evaluate stability of predictors while reducing overfitting. We performed 1,000 bootstrap resamples; in each resample, we refit the penalized model and selected the penalty parameter λ using 5‑fold cross‑validation, extracting coefficients at the cross‑validated optimum. Results are presented as mean odds ratios (mORs) and 95% bootstrap percentile confidence intervals across resamples. Because penalization can shrink coefficients to zero in some resamples, selection frequency may vary across bootstrap iterations.

### Sensitivity analyses

We explored eGFR < 75 as an outcome in sensitivity analyses, which were less-well powered given a small percentage of individuals with eGFR < 75. We also considered sex and age as effect measure modifiers in stratified sensitivity analyses.

## Results

Study participants in 2023 (*n* = 568) resided in five Nicaraguan regions (Table 1). Median age was 21 years (IQR = 7), 53% were female, and 41.9% (*n* = 238) had high BMI. Diabetes (*n* = 3; 0.5%), hypertension (*n* = 9; 1.6%), and prevalent CKD (0.35%; *n* = 2) were uncommon. Prevalence of urinary proteinuria from dipstick (≥ 1+) was 3.2% (*n* = 18; Table 1). Approximately 52% of participants (*n* = 288) were employed, primarily in service and professional roles (Table 2); 13.7% (*n* = 78) were employed in heat-intensive manual labor, including sugarcane production (*n* = 25; 4.4% overall), brickmaking (20; 3.5%), and construction (*n* = 13; 2.3%) (Table 2). Forty-nine percent (*n* = 280) of participants were students reporting no current or previous employment.

**Table 1:** Select characteristics of Jovenes study participants in Nicaragua, 2023 (*N* = 568)

Characteristics	Total (*N* = 568)	Males (*n* = 269)	Females (*n* = 299)
Age in years (mean, SD)	21.1 (4.4)	21.0 (4.5)	21.1 (4.2)
Residence in high-risk region^a^	411 (72.4)	192 (71.4)	219 (73.2)
Low socioeconomic status^b^	218 (38.4)	121 (45.0)	97 (32.4)
Pesticide use at home^c^	89 (25.7)	50 (18.6)	39 (13.0)
Residential drinking water source^d^			
Municipal source Other (includes well, river, bottled)	455 (80.1) 113 (19.9)	218 (81.0) 51 (19.0)	237 (79.3) 62 (20.7)
**Occupational exposures**, n(%)			
Heat-intensive manual laborer^e^	78 (13.7)	65 (24.2)	13 (4.3)
Sugarcane worker^f^	25 (4.4)	22 (8.2)	3 (1.0)
Brickmaking	20 (3.5)	16 (5.9)	4 (1.3)
Construction	13 (3.2)	9 (3.3)	4 (1.3)
Service jobs^g^	81 (14.2)		
Public and professional services^h^	129 (22.7)		
**Kidney health parameters**			
Estimated GFR (eGFR), mL/min/1.73m^2^ Mean (SD)^i^	101.5 (13.8)	99.0 (14.0)	103.8 (13.2)
eGFR < 90 mL/min/1.73m^2^, n (%)^j^	101 (17.8)	66 (24.5)	35 (11.7)
eGFR < 75 mL/min/1.73m^2^, n (%)^j^	23 (4.0)	17 (6.3)	6 (2.0)
eGFR < 60 mL/min/1.73m^2^, n (%)	6 (1.1)	4 (1.5)	2 (0.7)
MeN high risk profile, n (%)^k^	9 (1.6)	9 (3.3)	0 (0)
**Medical conditions**,** n (%)**			
High BMI^l^	238 (41.9)	113 (42.0)	125 (41.8)
Diabetes	3 (0.5)	2 (0.7)	1 (0.3)
Hypertension	9 (1.6)	5 (1.9)	4 (1.3)
Diagnosed CKD	2 (0.35)	1 (0.37)	1 (0.33)
Urinary proteinuria	18 (3.2)	10 (3.7)	8 (2.7)

^a^ High-risk regions include Chinandega and León; low-risk regions include Jinotega, Managua, Masaya, and Matagalpa

^b^ Low socioeconomic status defined as household size–to–income ratio < 0.35

^c^ Pesticide use at home refers to self‑reported pesticide application or the presence of pesticides in the home environment and does not represent occupational pesticide exposure

^d^ Primary drinking water source at home; other sources include private/public wells, river, and bottled water

^e^ Heat-intensive manual labor refers to occupations characterized by sustained physical exertion performed under conditions of elevated thermal load, including high ambient or radiant heat, limited convective cooling, and/or impaired heat dissipation

f Includes any primary or secondary work as laborers in sugarcane production from 2022–2023. Jobs in this category include seeders, cutters, and pesticide applicators

^g^ Service jobs include transportation, food service, shrimp processing, commercial textiles beauty services, home and family care/babysitting, house cleaning, municipal services, security surveillance, sports, and street vending

^h^ Employment in this category include banking and finance, commerce, customer service, health, education, entertainment, government, marketing and advertising, and the legal sector

^I^ eGFR was calculated using the European Kidney Function Consortium (EKFC) equation. eGFR categories are cumulative (i.e., < 90 includes < 75 and < 60)

^j^ *p* < 0.05 for comparisons between males and females

^k^ MeN high-risk profile defined as: eGFR < 75 mL/min/1.73m^2^ in participants without high BMI, diabetes, or hypertension

^l^ Body mass index (BMI) calculated as weight in kilograms divided by height in meters squared (kg/m²). High BMI defined as ≥ 85th percentile for age and sex, per CDC guidance for < 18 years, and > 24 for participants ≥ 18 years

Abbreviations: BMI, body mass index; CKD, chronic kidney disease; eGFR, estimated glomerular filtration rate; EKFC, European Kidney Function Consortium; IQR, interquartile range; MeN, Mesoamerican nephropathy; SD, standard deviation.

**Table 2:** Current employment or educational activity for Jovenes study participants, 2023 (N = 568)

Current employment or educational activity	*n* (%)
Not working for salary	280 (49.3)
Full-time student, not working^a^	157 (27.6)
Non-student, not working	96 (16.9)
Employed for salary	288 (50.7)
Hours worked per day	
1–3 4–6 7–9 10+	9 (3.1) 47 (16.3) 132 (45.8) 100 (34.7)
Heat-intensive manual laborers^b^	78 (13.7)
Agriculture Sugarcane^b^ Grain farming^b^ Animal herding^b^	33 (5.8) 25 (4.4) 7 (1.2) 1 (0.2)
Mining, manufacturing, and construction Brick making^b^ Construction^b^ Mining^b^	45 (7.9) 20 (3.5) 13 (2.3) 12 (2.1)
Service and labor Transportation Food service Shrimp processing Commercial textiles Beauty service Home and family care/ babysitting House cleaning Municipal services Security surveillance Sports Street vendor	81 (14.2) 16 (2.8) 14 (2.5) 7 (1.2) 24 (4.2) 5 (0.9) 5 (0.9) 3 (0.5) 2 (0.4) 2 (0.4) 2 (0.4) 1 (0.2)
Public and professional services Banking and finance Commerce Customer service Health Education Entertainment Government Marketing and advertising Legal	129 (22.7) 7 (1.2) 74 (13.0) 3 (0.5) 23 (4.0) 13 (2.3) 2 (0.4) 4 (0.7) 2 (0.4) 1 (0.2)

^a^ For the purpose of this analysis, 27 participants were considered employed for a salary that were part-time students and part-time workers

^b^ Heat-intensive manual laborers described as those working outdoors in high-heat environments with intense physical exertion over prolonged periods of time. These occupations include sugarcane, grain farming, animal herding, brickmaking, mining, and construction

### Low eGFR

**Fig. 1:**
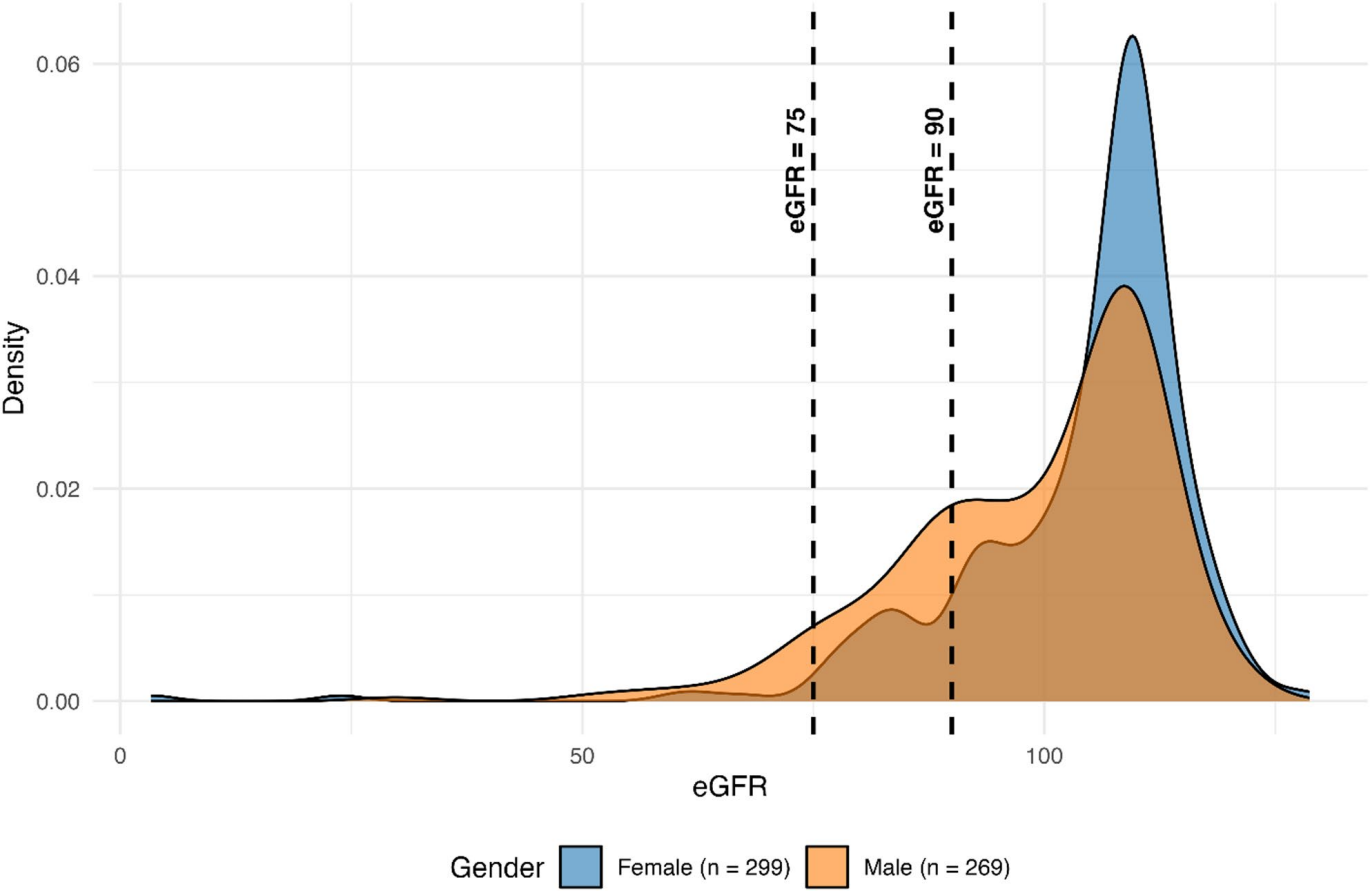
Estimated GFR distribution by gender in Jovenes participants, 2023 (*N* = 568). Distribution of eGFR by gender among participants living in high-risk regions for CKDu using the creatinine-based European Kidney Function Consortium (EKFC) equation. Dotted horizontal lines depict kidney function health endpoints with an eGFR < 90 ml/min/1.73m^2^ indicating reduced kidney function and more sensitive cutoff at eGFR < 75 ml/min/1.73m^2^

Median eGFR was 106.7 ml/min/1.73 m^2^ (IQR = 16.3) with no significance difference between males and females (Table 1; Fig. 1). Prevalence of eGFR < 90 ml/min/1.73 m^2^ was 17.8% (*n* = 101). Prevalence of eGFR < 75 ml/min/1.73 m^2^ was 4.1% (*n* = 23) and was significantly more common among males than females (6.3% vs. 2.0%; *p* = 0.001). Among individuals with eGFR < 75 ml/min/1.73 m^2^, 60.9% were non-working students and 39.1% (*n* = 9) were employed, five of whom worked in heat-intensive manual labor.

Prevalence of eGFR < 60 ml/min/1.73 m^2^ was 1.1% (*n* = 6; 2 females, 4 males) (Table 1). Median eGFR among these six individuals was 40.2 ml/min/1.73 m^2^ (IQR = 28.4). Five were not working, and one worked in a high-heat manual job (brickmaking).

### MeN high-risk profile

The MeN high-risk profile was observed in 1.6% (9 of 568) of participants, all male and all living in high MeN-risk regions (Table 3). Among this group, two had eGFR < 60 ml/min/1.73 m^2^ and reported a prevalent CKD medical diagnosis. Four were employed, including two heat-intensive manual laborers (sugarcane and brickmaking), and five were non-working students. Of 23 participants with eGFR < 75 ml/min/1.73 m^2^, nine fit the high-risk MeN profile (39.1%) (Table 1).

**Table 3:** Characteristics of individuals meeting the MeN high-risk profile, (*n* = 9)^a^

Characteristic	Total (*n* = 9); Mean (SD)
eGFR (EKFC), mL/min/1.73m^2^	66.7 (7.6)
Male sex	9 (100%)
BMI	20.6 (2.3)
Average age, years	19.2 (4.3)
Living in a high-risk region^b^	9 (100%)
Low socioeconomic status	4 (44.4%)
Municipal water source (ENACAL)	6 (66.7%)
Currently attending school	5 (55.6%)
**Currently employed**	4 (44.4%)
Heat‑intensive manual labor - sugarcane	1 (11.1%)
Heat‑intensive manual labor - brickmaking	1 (11.1%)
Pesticide use at work	0 (0%)
Urinary proteinuria	1 (11.1%)

^a^ The definition for the MeN high-risk profile is eGFR < 75 mL/min/1.73m^2^ and without reported hypertension, diabetes, or high BMI

^b^ High-risk regions were defined as areas with high levels of CKDu prevalence and included, La Paz Centro, Mina el Limon, Chinandega and low risk being Managua, Jinotega, and Masaya

### Regression analysis

**Fig. 2:**
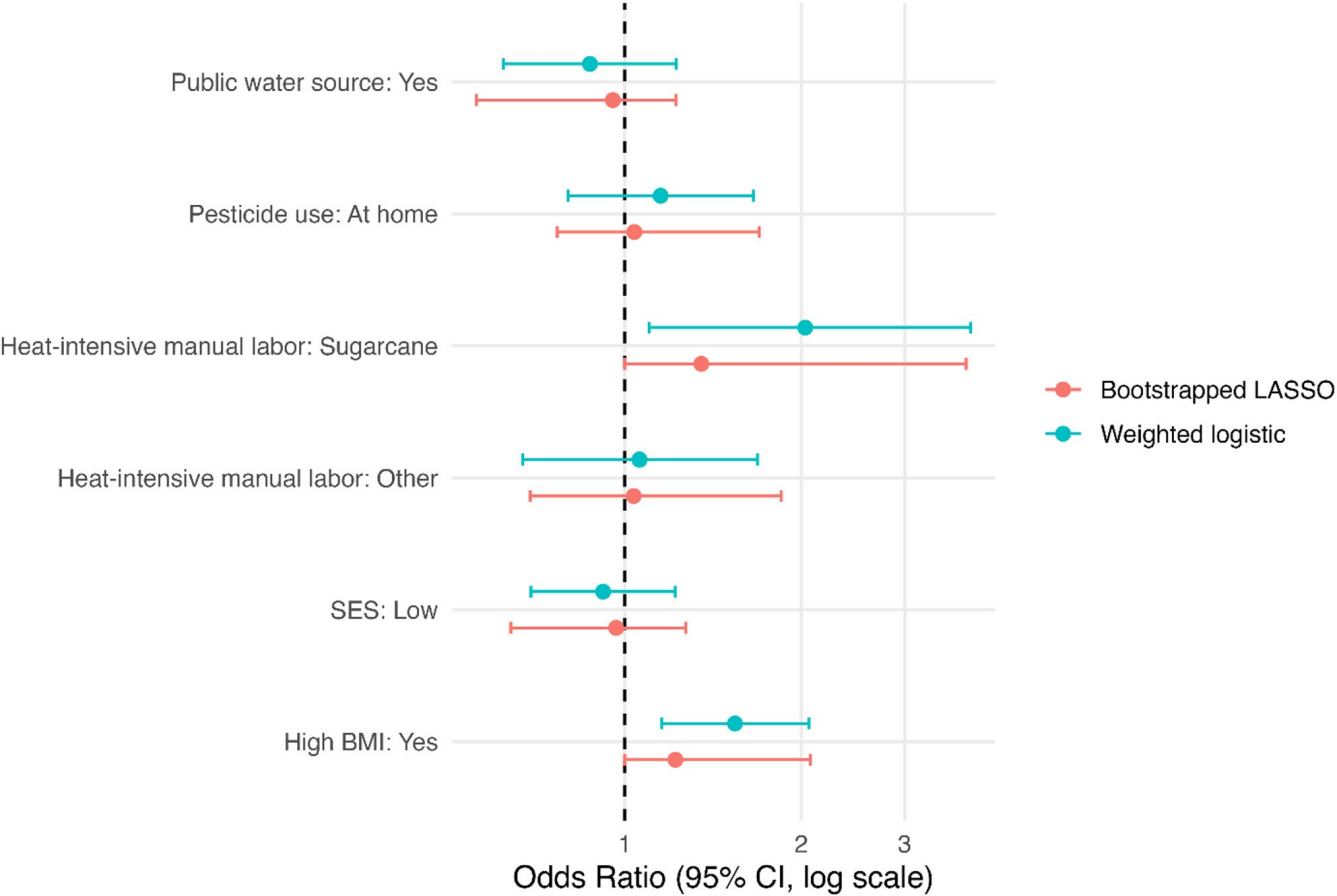
Predictors of eGFR < 90 mL/min/1.73m^2^ among Nicaraguan adolescents (*n* = 568), in two regression models. Forest plot of odds ratios and 95% confidence intervals on the log scale. Models were sex and age adjusted regression results predicting low eGFR among Jovenes participants. Dotted line represents the null value for both models. Sugarcane work and high BMI were consistent predictors of low eGFR in both models. The 95% confidence intervals of all other effect estimates include the null value and therefore were not significant predictors of low eGFR

Heat‑intensive manual labor in sugarcane was associated with eGFR < 90 in age- and sex-adjusted weighted logistic regression (aOR 2.03; 95% CI 1.10, 3.88) and bootstrapped LASSO‑penalized logistic regression (mOR 1.35; 95% CI 1.00, 3.82) (Fig. 2; Table 4). High BMI for age was positively associated with low eGFR in both models (aOR 1.54; 95% CI 1.16, 2.06) in the weighted regression model and (mOR 1.22; 95% CI 1.00, 2.07) using LASSO. Public water source, low SES, heat‑intensive manual labor in other industries (basic grains, construction, herding, mining, and clay/brick/tile work), and pesticide use at home were not significantly associated with eGFR < 90 (Fig. 2).

**Table 4:** Weighted and LASSO regression results with low eGFR outcome (< 90 ml/min/1.73m^2^)^1^

	Weighted logistic regression model (OR, 95% CI)	LASSO Regression (Mean OR, 95% CI)
Age (per 1-year increase) year	0.97 (0.94, 1.00)0.97	(0.92, 1.03)
Sex	2.29 (1.73, 3.02)	2.39 (1.47, 3.85)
Municipal water source^2^	0.87 (0.62, 1.22)	0.96 (0.56, 1.22)
Pesticide use at home^3^	1.15 (0.80, 1.66)	1.04 (0.77, 1.70)
Low socioeconomic status^4^	0.92 (0.69, 1.22)	0.97 (0.64, 1.27)
Heat‑intensive manual labor in the sugarcane^5^	2.03 (1.10, 3.88)	1.35 (1.00, 3.82)
Heat‑intensive manual labor in other industries^5^	1.06 (0.67, 1.69)	1.04 (0.69, 1.85)
High BMI^6^	1.54 (1.16, 2.06)	1.22 (1.00, 2.07)

^1^ Models were adjusted for sex and age and independent estimates from those covariates were not interpreted. Bootstrapped LASSO estimates were generated from 1,000 bootstrap iterations; in each resample, a LASSO-penalized logistic regression model was fit with 5-fold cross-validation to select λ, and results are presented as the mean odds ratio (mean OR) with 95% bootstrap percentile confidence intervals

^2^ Municipal water source defined dichotomously as “ENCAL” (public water utility) or other (well, river, bottled)

^3^ Pesticide use at home was self-reported in questionnaire, with insufficient participants reporting occupational pesticide exposure (*n* = 1) to evaluate this exposure

^4^ Low SES was defined as a binary indicator derived from the household SES ratio (workers/household size), dichotomized at the median of the observed distribution, 0.35

^5^ Heat-intensive manual labor was defined using a mutually exclusive three-level exposure based on primary industry: “Sugarcane” (sugarcane industry), “Other” (basic grains/agriculture, construction, herding, mining, and clay/brick/tile work), and “No” (not employed in these heat-intensive industries); the reference group for heat-intensive manual labor comparisons was “No”. Sex was coded as 0 = female and 1 = male; the sex odds ratio compares males versus females. Age was modeled continuously (years)

^6^ High BMI defined as ≥ 85th percentile for age and sex, per CDC guidance for < 18 years [37], and > 24 for participants ≥ 18 years

## Discussion

We assessed the prevalence of low eGFR and a high-risk MeN profile among male and female adolescents and young adults in Nicaragua, an understudied group in a region heavily impacted by CKDu. We observed five key findings: (1) elevated prevalence of eGFR < 75 and < 60 mL/min/1.73 m² among Nicaraguan adolescents compared to international referents; (2) heightened prevalence of high BMI and the positive association between high BMI and low eGFR; (3) low eGFR among adolescent girls and young women, notable in a region where disease presentation is male-dominated; (4) heat‑intensive sugarcane work as a risk factor for low eGFR; and (5) low eGFR among youth without occupational heat exposure. Each finding is discussed below.

Our study participants experience elevated prevalence of moderate to severe kidney dysfunction compared to international referents. Assessments of U.S. adolescents indicate that while eGFR < 90 mL/min/1.73 m² affects approximately one-third of adolescents, eGFR < 75 mL/min/1.73 m² is uncommon (1–2%), and eGFR < 60 mL/min/1.73 m² is rare (< 0.5%), with equal distribution by sex [38]. Our prevalences at these lower thresholds are 2-3x higher than these U.S. referents. Our estimates likewise exceed those from a Malaysian school-based cohort [39] but correspond with reports from India in which approximately 5% of youth experienced eGFR < 60 mL/min/1.73 m² [40]. 

While high BMI is an established risk factor for progressive kidney dysfunction in youth, it is uncommon in occupational CKDu studies involving adults [3, 32, 41–45]. Our data suggest that young people in this region may face elevated risk of both metabolic and non-metabolic kidney disease. We distinguished a high-risk MeN profile to exclude individuals with metabolic risk factors and observed that all individuals meeting this profile were male. BMI-related renal impairment does not preclude other environmental, genetic, or occupational risks for kidney disease, which remain important areas for continued research.

Girls and young women in Central America are not typically included in MeN research because case presentation is male dominated [46]. Our findings indicate that adolescent girls and young women may experience clinically relevant low eGFR (e.g. 2.0% of females with eGFR < 75 mL/min/1.73 m²). All females with low eGFR at this threshold had comorbid high BMI and therefore did not meet the high-riskMeN profile. Our data suggest that metabolic factors may play a key role in kidney dysfunction among girls and young women in this population. Although females did not fit the MeN high-risk profile, renal impairment among females prompts broadening surveillance and screening efforts beyond agricultural workers to address interventions relevant to kidney health, including nutritional, dietary, and behavioral interventions.

Heterogenous profiles support a multifactorial disease model. Our data indicates at least two patterns: (1) a non-metabolic profile consistent with early MeN risk, occurring among males and associated with heat exposure; and (2) metabolic kidney impairment, more commonly observed among females and BMI-associated. The coexistence of these profiles suggests that both MeN and metabolic pathways may be active in adolescence, with individual environmental and social context shaping individual trajectories.

We observed a significant relationship between sugarcane employment and eGFR < 90 mL/min/1.73 m², consistent with other studies [1, 8, 41, 43, 47–50]. However, most participants in our study with eGFR < 75 mL/min/1.73 m² (> 90%) and the MeN profile (> 87%) did not engage in heat-intensive manual labor. These results suggest that while occupational heat stress may contribute to early kidney function decline in specific subgroups, it does not fully explain the broader burden of reduced eGFR observed in this adolescent and young adult population. Our findings parallel studies of youth in CKDu-impacted regions in which biomarkers of acute kidney injury biomarkers are observed among youth not yet in the workforce [13, 14, 18]. 

Strengths of this analysis include the adolescent and young adult age range, diversity of occupational and non-occupational activities, large sample size, and inclusion of both genders. Limitations include underrepresentation of high-heat and pesticide-exposed workers which limit our ability to consider those occupational risk factors comprehensively. Kidney function was assessed using a single measurement at the 2023 study visit, which may result in misclassification of eGFR; repeat testing within a one-year period was not feasible within the constraints of our community-based study design. Although this approach limits clinical confirmation of chronic kidney dysfunction, it is consistent with population-based epidemiologic studies in similar settings and underscores the need for longitudinal follow-up to better characterize persistence and progression of kidney abnormalities in this population. Because serum creatinine was measured at a single time point, we were unable to distinguish transient reductions in kidney function from persistent impairment, which may lead to misclassification of kidney health status. Future work could incorporate repeated measures to distinguish chronic from acute changes and to better define trajectories of kidney function over time. The use of an eGFR threshold of < 75 (vs. 60 or 90) as a descriptive risk marker is less aligned with KDIGO standards, but consistent with recent literature on kidney health among adolescent and young adult populations; due to relatively small counts, we were unable to explore risk factors for this threshold using regression modeling, which is a limitation [51]. Exposure assessment in this analysis relied on self‑reported occupational and household histories, which may be subject to recall bias and limit the precision of exposure categorization. Although we categorized heat intensive manual labor using detailed interviews, short term, seasonal, or informal work may be underreported, and cumulative childhood participation in family agriculture may not be fully captured, potentially biasing heat exposure effect estimates toward the null.

Finally, although our sample included participants from multiple regions in the country, some subgroups, particularly sugarcane workers and individuals with severe kidney dysfunction, were small, reducing the statistical power to detect modest associations and limiting generalizability across CKDu‑affected settings. Additionally, our findings may reflect undiagnosed congenital or structural renal abnormalities affecting kidney health in adolescence and young adulthood that are beyond the scope of this study.

## Conclusion

We observed elevated prevalence of low eGFR among Nicaraguan adolescents and young adults, including among females, those both with and without occupational heat stress exposure, and those with and without metabolic risk factors. Our findings suggest early and heterogenous pathways to kidney dysfunction in this region not fully explained by occupational exposures.

## Supplementary Information

Below is the link to the electronic supplementary material.

Supplementary Material 1

Supplementary Material 2

## Data Availability

The datasets generated and analyzed during this current study are publicly available at: https://github.com/yiryuan/Kidney_health_among_adolescents_living_in_a_high-risk_region.
