# Supplementary material for: Kidney health among adolescents living in a high-risk region for CKDu
Source: BMC Nephrol. 2026 Jun 4;27:483. doi: 10.1186/s12882-026-05070-1 (PMC13483704; doi:10.1186/s12882-026-05070-1)
Supplement: Supplementary file 1 [file 12882_2026_5070_MOESM1_ESM.docx]

Supplemental Materials

Laboratory procedures: Urine specimens were assessed for specific gravity, pH, leukocytes, nitrites, proteins, glucose, ketones, urobilinogen, bilirubin, blood, hemoglobin, and a urine dipstick analysis at Centro Médico del Pacifico S.A. (CENMED) Laboratory using Roche Urysis 1100 with Combur 10 UX reactive strips. Urine concentration was assessed in the field using a handheld digital refractometer (model and manufacturer), which provides urine specific gravity (USG). Osmolality was estimated from USG using standard empirical relationships under typical physiologic solute ranges. Serum was analyzed at CENMED laboratories for rapid report-back of creatinine levels to study participants (within 2 weeks of participation) and then again at Quest Diagnostics in Boston, MA using the basic renal function panel (albumin, BUN/creatinine Ratio, calcium, carbon dioxide, chloride, creatinine, glucose, phosphorus, potassium, sodium, and urea nitrogen (BUN). Data from Quest Diagnostics were used for the analyses in this study.

Statistical approach: Serum albumin, BUN/creatinine ratio, calcium, creatinine, glucose, hematocrit, phosphorous, potassium, sodium and urea nitrogen were assessed and summarized. Urine parameters chloride, total CPK, hematocrit, specific gravity, and urine osmolality were also assessed and summarized.

**Table S1.** Renal health parameters by sex and MeN phenotype among Jovenes study participants in Nicaragua, 2023

| **Parameter** | **Overall**  **(N=568)** | **MeN**  **Phenotype**  **(n=9)** | **Males (n=269)** | | **Females (n=299)** | |
| --- | --- | --- | --- | --- | --- | --- |
|  |  |  | **Males**  **eGFR ≥90**  **(n= 203)** | **Males**  **eGFR <90**  **(n= 66)** | **Females**  **eGFR ≥90**  **(n= 264)** | **Females**  **eGFR <90**  **(n= 35)** |
| **Median (IQR)** | | | | | | |
| **Blood urea nitrogen (BUN) (mg/dL)** | 9.0 (3.0) | 14.0 (5.0) | 9.0 (3.0) | 11.0 (4.0)* | 8.0 (3.0) | 11.0 (4.5)* |
| **Total CPK (U/L)** | 114.9(106.2) | 183.4 (145.1) | 153.0 (110.8) | 193.6 (165.1)* | 81.2 (38.2) | 122.9 (153.9)* |
| **Creatinine (mg/dL)** | 0.8 (0.2) | 1.2 (0.2) | 0.9 (0.2) | 1.1 (0.1)* | 0.7 (0.1) | 0.9 (0.1)* |
| **Sodium (mmol/L)** | 137.0 (3.0) | 136.0 (4.0) | 137.0 (2.0) | 137.0 (2.8) | 136.0 (2.0) | 137.0 (1.0) |
| **Uric acid (mg/dL)** | 5.0 (2.0) | 8.3 (2.8) | 5.7 (1.6) | 7.0 (1.7)* | 4.2(1.5) | 4.8 (1.4)* |
| **Albumin (g/dL)** | 4.5 (0.3) | 4.6 (0.5) | 4.7 (0.3) | 4.7 (0.3) | 4.4 (0.3) | 4.4 (0.3) |
| **Calcium (mg/dL)** | 9.5 (0.5) | 9.6 (0.6) | 9.6 (0.5) | 9.6 (0.5) | 9.4 (0.5) | 9.3 (0.5) |
| **Chloride (mmol/L)** | 102.0 (3.0) | 100.0 (2.0) | 102.0 (3.0) | 101.0 (3.0) | 103.0 (3.0) | 103.0 (3.0) |
| **Glucose (mg/dL)** | 88.0 (14.0) | 94.0 (24.0) | 88.0 (15.0) | 89.0 (14.2) | 88.0 (13.2) | 84.0 (15.0) |
| **Hematocrit (%)** | 40.0 (4.0) | 41.0 (3.0) | 43.0 (5.0) | 43.0 (5.8) | 39.0 (3.0) | 39.0 (2.5) |
| **Phosphate (mg/dL)** | 4.0 (0.8) | 3.9 (0.7) | 4.1 (0.8) | 4.0 (0.8) | 3.9 (0.7) | 3.7 (0.5) |
| **Potassium (mmol/L)** | 4.0 (0.3) | 3.7 (0.7) | 4.0 (0.4) | 3.9 (0.3) | 4.0 (0.3) | 3.8 (0.5) |
| **Specific gravity** | 1.015 (0.010) | 1.015 (0.010) | 1.015(0.005) | 1.015 (0.010) | 1.015 (0.010) | 1.020 (0.010) |
| **Urine osmolality (mOsm/kg)** | 620.0 (380.0) | 500.0 (480.0) | 640.0 (397.5) | 630.0 (440.0) | 620.0 (365.0) | 640.0 (450.0) |

***Indicates significant differences between sex-specific eGFR groups at P <0.05.

All eGFR calculations were made using the European Kidney Function Consortium (EKFC) equation; a race and gender free equation validated for ages ≥2 years old. Continuous variables are reported in median and standard deviation, and binary outputs are reported as totals and percentages. Further data may not be added to 100% in all columns due to missing responses. Urine parameters were analyzed at Centro Médico del Pacifico S.A. (CENMED) Laboratory in Masaya, Nicaragua, using Roche Urysis 1100 with Combur 10 UX reactive strips. Blood parameters were analyzed at Quest Diagnostics in Boston.
