# Supplementary material for: Kidney health among adolescents living in a high-risk region for CKDu
Source: BMC Nephrol. 2026 Jun 4;27:483. doi: 10.1186/s12882-026-05070-1 (PMC13483704; doi:10.1186/s12882-026-05070-1)
Supplement: Supplementary file 2 [file 12882_2026_5070_MOESM2_ESM.docx]

**Participant ID: ____________________________**

***YOUTH SURVEY QUESTIONNAIRE, ROUND 2: 2023***

| Entry completed | ☐ | ☐ |
| --- | --- | --- |
|  | YES | NO |

**Georeferencing points**

Length: ______________

Latitude: _______________

*INFORMATION FROM THE INTERVIEWER:*

*Interviewer’s initials: Date of interview _*___/____/_____

Day Month Year

Interview start time: ______ (Please enter this into military time)

*ENTRY OF DATABASE INFORMATION:*

Quality control initials: Date of *entry ____/____/_*____

Day/ Month/ Year

**Demographic information**

1) Participant phone number: ________

2) Biological sex

☐ Male

☐ Female

3) Age: ________

**Community, Residence, and Education**

*Thank you for joining our study. We appreciate your participation in this research! During today’s visit, we would like to ask you some questions about yourself, your home, your work and health.*

1) In which **department** do you live now?

| ☐ | ☐ | ☐ | ☐ | ☐ | ☐ |
| --- | --- | --- | --- | --- | --- |
| Chinandega | León | Masaya | Jinotega | Managua | Other (Specify) |

2) In which **municipality** do you live now?

| ☐ | ☐ | ☐ | ☐ | ☐ | ☐ | ☐ |
| --- | --- | --- | --- | --- | --- | --- |
| Chichigalpa | Jinotega | La Paz Centrol | Mina El Limón | Managua | Masaya | Other (Specify) |

3) Have you moved to a new community since we last spoke with you?

☐ Yes (Please write your new address): ____________

☐ NO

4) Select your current student status that most applies to you

☐ I attend school full time

☐ I attend school part time

☐ I do not attend school at present

Answer only IF “I ATTEND SCHOOL FULL TIME”

4a) What is your current academic level?

| ☐ | ☐ | ☐ |
| --- | --- | --- |
| High school | Technical school | University |

5) Are you working now?

☐ YES

☐ NO

5a) Do you receive a salary or payment for your work now?

☐☐ YES

☐ NO

5b) IF YES to question 6: Is this your first job?

☐ YES

☐ NO

5b) IF YES to question 6: Did you begin this job after we met you last year?

☐ YES

☐ NO

6) Has any aspect of your employment changed since we met you last year?

☐ YES

☐ NO

6b) IF YES to question 8: Please describe the changes:

| ☐ | ☐ | ☐ | ☐ |
| --- | --- | --- | --- |
| I moved and changed jobs | I was promoted | I started working more hours | I started working less hours |
| ☐ | ☐ | ☐ | ☐ |
| My job tasks have changed | I lost my job | I have more on the job responsibilities | Other (please explain): |

7) IF YES to question 6a (Interviewer, please read options out loud): Please fill in everything that applies to your **current** job.

| Industry | What is or was your job in the activities you performed ? | Do you work part-time while also attending school? | Did you work full year or some months of year? | How many hours a day did you work (on this job)? | Since 2022, how many months did you work?  (even if it is intermittent) |
| --- | --- | --- | --- | --- | --- |
| ☐Sugar cane agriculture |  | ☐ YES  ☐ NO | ☐ Some months of the year  ☐ All year | ☐ 1-3 ☐ 4-6  ☐ 7-9 ☐ 10+ | ☐ 1-3 ☐ 4-6  ☐ 7+ |
| ☐ Food service |  | ☐ YES  ☐ NO | ☐ Some months of the year  ☐ All year | ☐ 1-3 ☐ 4-6  ☐ 7-9 ☐ 10+ | ☐ 1-3 ☐ 4-6  ☐ 7+ |
| ☐Basic Grain Farming |  | ☐ YES  ☐ NO | ☐ Some months of the year  ☐ F All year | ☐ 1-3 ☐ 4-6  ☐ 7-9 ☐ 10+ | ☐ 1-3 ☐ 4-6  ☐ 7+ |
| Home and family assistant, nanny |  | ☐ YES  ☐ NO | ☐ Some months of the year  ☐ All year | ☐ 1-3 ☐ 4-6  ☐ 7-9 ☐ 10+ | ☐ 1-3 ☐ 4-6  ☐ 7+ |
| ☐ Shrimp boat |  | ☐ YES  ☐ NO | ☐ Some months of the year  ☐ All year | ☐ 1-3 ☐ 4-6  ☐ 7-9 ☐ 10+ | ☐ 1-3 ☐ 4-6  ☐ 7+ |
| ☐ Trade |  | ☐ YES  ☐ NO | ☐ Some months of the year  ☐ All year | ☐ 1-3 ☐ 4-6  ☐ 7-9 ☐ 10+ | ☐ 1-3 ☐ 4-6  ☐ 7+ |
| ☐Construction |  | ☐ YES  ☐ NO | ☐ Some months of the year  ☐ All year | ☐ 1-3 ☐ 4-6  ☐ 7-9 ☐ 10+ | ☐ 1-3 ☐ 4-6  ☐ 7+ |
| ☐ Cabinetmaking |  | ☐ YES  ☐ NO | ☐ Some months of the year  ☐ All year | ☐ 1-3 ☐ 4-6  ☐ 7-9 ☐ 10+ | ☐ 1-3 ☐ 4-6  ☐ 7+ |
| ☐ Cattle raising |  | ☐ YES  ☐ NO | ☐ Some months of the year  ☐ All year | ☐ 1-3 ☐ 4-6  ☐ 7-9 ☐ 10+ | ☐ 1-3 ☐ 4-6  ☐ 7+ |
| ☐ Mining Industry |  | ☐ YES  ☐ NO | ☐ Some months of the year  ☐ All year | ☐ 1-3 ☐ 4-6  ☐ 7-9 ☐ 10+ | ☐ 1-3 ☐ 4-6  ☐ 7+ |
| ☐ Mud industry (bricks and tiles) |  | ☐ YES  ☐ NO | ☐ Some months of the year  ☐ All year | ☐ 1-3 ☐ 4-6  ☐ 7-9 ☐ 10+ | ☐ 1-3 ☐ 4-6  ☐ 7+ |
| ☐ Health sector |  | ☐ YES  ☐ NO | ☐ Some months of the year  ☐ All year | ☐ 1-3 ☐ 4-6  ☐ 7-9 ☐ 10+ | ☐ 1-3 ☐ 4-6  ☐ 7+ |
| ☐ Education Sector |  | ☐ YES  ☐ NO | ☐ Some months of the year  ☐ All year | ☐ 1-3 ☐ 4-6  ☐ 7-9 ☐ 10+ | ☐ 1-3 ☐ 4-6  ☐ 7+ |
| ☐ Security Surveillance |  | ☐ YES  ☐ NO | ☐ Some months of the year  ☐ All year | ☐ 1-3 ☐ 4-6  ☐ 7-9 ☐ 10+ | ☐ 1-3 ☐ 4-6  ☐ 7+ |
| ☐ House cleaning |  | ☐ YES  ☐ NO | ☐ Some months of the year  ☐ All year | ☐ 1-3 ☐ 4-6  ☐ 7-9 ☐ 10+ | ☐ 1-3 ☐ 4-6  ☐ 7+ |
| ☐ Transporting |  | ☐ YES  ☐ NO | ☐ Some months of the year  ☐ All year | ☐ 1-3 ☐ 4-6  ☐ 7-9 ☐ 10+ | ☐ 1-3 ☐ 4-6  ☐ 7+ |
| ☐ Textiles- Free Trade Zone |  | ☐ YES  ☐ NO | ☐ Some months of the year  ☐ All year | ☐ 1-3 ☐ 4-6  ☐ 7-9 ☐ 10+ | ☐ 1-3 ☐ 4-6  ☐ 7+ |
| Street vendor |  | ☐ YES  ☐ NO | ☐ Some months of the year  ☐ All year | ☐ 1-3 ☐ 4-6  ☐ 7-9 ☐ 10+ | ☐ 1-3 ☐ 4-6  ☐ 7+ |
| ☐ Other |  | ☐ YES  ☐ NO | ☐ Some months of the year  ☐ All year | ☐ 1-3 ☐ 4-6  ☐ 7-9 ☐ 10+ | ☐ 1-3 ☐ 4-6  ☐ 7+ |

**Health history**

*Next, we want to ask you questions about your health.*

1) In the last year, have you had health problems that have affected your ability to work or attend school (either diagnosed or undiagnosed by a doctor or nurse)?

☐ YES

☐ NO

☐ I don’t know

1a) IF YES to question 1 (Interviewer please read options out loud): Please specify which health-related problems newly affected your work:

| ☐ Creatinine elevation | ☐  Low potassium (hypopokalemia) | ☐  Kidney stones | ☐  Complications due to an infectious disease (please specify) | ☐ Musculoskeletal disorders | ☐  Dental Diseases | ☐  Pregnancy complications | ☐  Skin lesions (e.g., Keratosis, melanosis, hyperpigmentation) | |
| --- | --- | --- | --- | --- | --- | --- | --- | --- |
| ☐  Neurological problems | ☐  Mental health | ☐ Autoimmune disorder | ☐  Heart problems | ☐  Diabetes | ☐ Hypertension | ☐  Inflammation | ☐  Kidney disease (CKD) | ☐  Other  (please specify) |

2) Would you say that in general your health is (Interviewer please read options out loud):

| ☐ | ☐ | ☐ | ☐ | ☐ | ☐ |
| --- | --- | --- | --- | --- | --- |
| Excellent | Very Good | Good | Fair | Poor | I don’t know |

3) Now thinking about your physical health, which includes physical illness and injury, for how many days during the past 30 days was your physical health not good?

☐ none

☐ Number of days: _____

☐ I don’t know

4) How many times in the last 30 days have you altered your lifestyle because of your physical health

☐ Number of days: _____

☐ None

☐ not sure

5) Regarding your mood, during the past 2 weeks, how often have you been bothered by feeling down, depressed, or hopeless?

☐ Once in a while

☐ Sometimes

☐ Never

6) Regarding your mood over the past two weeks, how often have you been bothered by feeling down, depressed, or hopeless?

☐ Once in a while

☐ Sometimes

☐ Never

7) In the last year, have you visited a doctor or nurse for kidney problems?

☐ YES

☐ NO

☐ I don’t know

8) Has a doctor or nurse ever told you that you suffer from kidney problems?

☐ YES

☐ NO

☐ I don’t know

8a) **If** **YES to 8**: What YEAR did they tell you? _________

8b) **If** **YES to 8**: Please specify what the nurse or doctor told you: _________

8c) **If** **YES to 8**: Did you receive a treatment plan?

☐ YES

☐ NO

☐ I don’t know

8c1) **If** **YES to 6:** Have you been able to meet your treatment plan?

☐YES

☐ NO

☐ I don’t know

8b2) **If** **YES to 8**: Please describe your treatment plan. *Please check all that apply*

| ☐ | ☐ | ☐ | ☐ | ☐ | ☐ | ☐ | ☐ | ☐ |
| --- | --- | --- | --- | --- | --- | --- | --- | --- |
| Take iron | Erythropoietin injection | Change of diet and meals. | Consumption of vitamins and supplements | Uses diuretics | Hemodialysis | Peritoneal dialysis | Allopurinol | Other:  (please specify) |

9) Have you ever been diagnosed with any of the following conditions (Interviewer, please read options out loud)? (*Interviewer please write legibly)*

*(If your chronic condition is not on the list, describe it in the section listed as* *other)*

☐ Does not apply

| **Conditions** | **Treatment (***Specify any treatment you receive or use for these conditions, including medicines and herbal remedies)* | **At what age were you when you were diagnosed? (in years)** |
| --- | --- | --- |
| ☐ Chronic Kidney Disease |  |  |
| ☐ Systemic Arterial Hypertension (HTA) |  |  |
| ☐ Type 1 diabetes |  |  |
| ☐ Type 2 diabetes |  |  |
| ☐ Heart Problems |  |  |
| ☐ Obesity/ being overweight |  |  |
| ☐ Anemia |  |  |
| ☐ Kidney stones |  |  |
| ☐ Liver disease |  |  |
| ☐ Chronic obstructive pulmonary disease |  |  |
| ☐ Asthma |  |  |
| ☐ Skin lesions ( e.g.s Keratosis, melanosis, hyperpigmentation |  |  |
| ☐ Parasites  (Specify):  ☐ Entamoeba  ☐ Histolytic  ☐ Giardia lamblia  ☐ Cryptosporidium |  |  |
| ☐ Stroke |  |  |
| ☐ OTHER: Please describe | |  |
| ☐ None of these | |  |

10) Have you been hospitalized since we last spoke with you (spent a night or more in the hospital)? For women, do not include hospitalizations for healthy childbirth.

☐YES

☐ NO

☐ I don’t know

10a) IF YES: Please fill in the following tables:

Hospitalization 1

☐ Age at time hospitalization: _______

Year

☐ 2022

☐ 2023

☐ 2024

Reason for hospitalization (briefly describe, *interviewer please write legibly)*

| ☐ Accident or injury | ☐ Infectious disease (specify which, if known) | ☐ Chronic condition  *Please specify condition below* | ☐ Pregnancy complications | ☐ Other: (Please specify) |
| --- | --- | --- | --- | --- |
|  |  |  |  |  |

Hospitalization 2

☐ Age of hospitalization: _______

Year

☐ 2022

☐ 2023

Reason for hospitalization (briefly describe, *interviewer please write legibly)*

| ☐ ☐Accident or injury | ☐ Infectious disease (specify which, if known) | ☐ Chronic condition | ☐ Pregnancy complications | ☐ Other: (Please specify) |
| --- | --- | --- | --- | --- |
|  |  |  |  |  |

11) Do you take any medications once a week or more?

☐ YES

☐ NO

☐ I don’t know

11a) IF YES: **What is the medicine(s) you take?**

**Medication**

☐ Analgesics

☐ Acetaminophen (Paracetamol)0

☐ Antibiotics (Amikacin, Gentamicin)

☐ Antihypertensives (Enalapril, Losartan, Nifedipine)

☐ Insulin, Metformin, or other Hypoglycemics

☐ Antifungals (Fluconazole, Ketoconazole)

☐ Diuretics (Furosemide, Thiazides)

☐ Urinary Antiseptics (Phenazopyridine, Pyridium)

☐ NSAIDs (Ibuprofen, Meloxicam, Indomethacin, Diclofenac, Voltaren, Ketoprofen)

☐ Immunosuppressants (Methotrexate, Plaquenil)

☐ Potassium Supplements (Ionak, Corpotasin)

☐ Multivitamins (including prenatal vitamins) (Folic Acid Supplements, Iron Supplements)

☐ Allopurinol

☐ Other (explain)

12) Since we met you last, have you used home remedies or herbal medicines?

☐ YES

☐ NO

☐ I don’t know

12a) If I answer YES to question 12 list the home remedies or herbal remedies you normally use at least once a month and the(s) reason(s) for their use. If you answered *NO,* go to question 11

| Remedy | How its prepared? | The reason for use  (Interviewer please write legibly) | Frequency of use (daily, at least weekly, at least monthly, less than monthly) | Used in the last 3 months? | Used in the last year | Where did you learn how to use this/ prepare this? |
| --- | --- | --- | --- | --- | --- | --- |
|  |  |  | ☐ Daily  ☐ At least once per week  ☐ At least once per month  ☐ Less than once per month | Yes \| No | ☐ Yes \| ☐ No |  |
|  |  |  | ☐ Daily  ☐ At least once per week  ☐ At least once per month  ☐ Less than once per month | Yes \| No☐ | ☐ Yes \| ☐ No |  |
|  |  |  | ☐Daily  ☐ At least once per week  ☐ At least once per month  ☐ Less than once per month | Yes \| No☐ | ☐ Yes \| ☐ No |  |

13) Do you take any medications or botanical remedies?

(By this, we refer to processed commercial plant-based preparations, such as capsules, powders, syrups, etc.)

☐ YES (Which ones): _____________

☐ NO

**Urination and Infections**

*In the next section, we will ask questions related to urination and infections.*

1. Have you ever experienced chistata – painful urination?

- YES
- NO
- I don’t know

**If NO: MOVE TO NEXT SECTION (UTI)**

**IF YES to 1:**

2. How old were you when you first experienced chistata? ________years old
3. Have you experienced chistata in **the last year**?

- YES
- NO
- I don’t know

4. Have you had chistata in the **last 48 hours?**

- YES
- NO
- I don’t know

**IF YES: Pivot to chistata surveillance questionnaire. RESUME on Page 12**

5. Would you describe your experiences with chistata as chronic (happening all the time or most of the time) or episodic (happening only at specific times)?

- Chronic
- Episodic
- I don’t know

6. **If EPISODIC**: When do you typically experience chistata (select all that apply)?

- In the summer
- In the winter
- When I do certain activities (please specify):______________________
- For women: Along with my menstrual cycle
- Other pattern: (please specify): _____________
- There is no pattern in my chistata episodes

7. **If EPISODIC**: How long does your chistata typically last?

- Less than one day
- 2-5 days
- 5 or more days
- Other – specify____________________________

8. Does chistata prevent you from doing activities that you want to or need to do?

- Yes
- No
- I don’t know

9. Which of the following describes how chistata feels to you? Choose all that apply.

| ☐ Pain in my bladder before urination | ☐ Pain/burning only at the start of urination | ☐ Pain/burning sensation from the moment I start urinating until I finish. | ☐ Pain or burning sensation remaining after urinating |
| --- | --- | --- | --- |
| ☐ Constant pain, even when I am not urinating. | ☐ The pain/burning feels like a sudden sensation. | ☐ The pain feels like a sensation of aching or cramps. | ☐ The pain feels like a burning sensation. |
| ☐ It makes my genital area itch. | ☐ The pain comes and goes unpredictably. | ☐ The pain occurs at predictable times (please describe). | ☐ A sensation as if I cannot empty my bladder, even after urinating. |
| ☐ A sensation that I have to urinate constantly and urgently. | Other (Please describe): ______________________________ | | |

10. When you have chistata, where in your body do you feel pain?

- Pain in the genital area (urethra).
- Pain in the suprapubic area (bladder).
- Pain in the right or left flank (ureters).
- Pain in the renal lumbar region.
- Other area (Please Describe): _______

11. On a scale from 1-5, with 5 being the most pain imaginable, how painful is chistata to you?

12. Describe how the odor of your urine is perceived during an active episode of chistata: ______
13. What color is your urine when you are having an active episode of chistata? (Please circle)

14. When you have chistata, is your urine ever cloudy or foamy?
    - YES
    - NO
    - I don’t know
15. Have you ever had blood in your urine? (For women: do not include blood in the urine sample while menstruating.)
    - YES
    - NO
    - I don’t know

**If YES:** How often do you have blood in your urine?

- Daily
- Weekly
- Monthly
- Less than monthly
- I don’t know

**IF YES**: Do you have it at the same time as when you experience chistata?

- YES
- NO
- Sometimes
- I don’t know

**RESUME HERE FROM CHISTATA Q for participants with chistata in last 48 hours:**

16. Do other people in your family experience chistata?
    - YES
    - NO
    - I don’t know
17. Is there anything else you would like to tell us about your experiences with chistata? __________________________

**Questions on UTI**

18. Have you ever had a urinary tract infection (UTI) diagnosed by a doctor?
    - YES
    - NO
    - I don’t know

**If no or IDK, skip and continue….**

**IF YES: Do you have a UTI at the same time that you have chistata?**

- YES
- NO
- I don’t know

**IF YES: Does the experience of UTI differ from the experience of chistata?**

- YES
- NO
- I don’t know

IF YES: How does a UTI differ from chistata to you?

______________________________________________

19. **IF YES**: Do you have a UTI right now??
    - YES
    - NO
    - I don’t know
20. During an episode of chistata or a UTI, how many times do you get up at night to urinate?
    - 1
    - 2
    - 3
    - 4+
    - I don’t remember
21. On a typical night, how many times a night do you get up to urinate?
    - Never/ not usually
    - 1
    - 2
    - 3
    - 4+
    - I don’t remember
22. Do you typically have to hold your urine for extended periods and wait to use the bathroom until you are done with work or school?
    - YES
    - NO
    - I don’t know
23. If YES to question 23: Why do you hold your urine for extended periods of time? (select all that apply)

- Restroom too far from work site
- I don’t have permission to use the restroom
- No access to a restroom
- Restroom is not clean
- The restroom lacks water, toilet paper, or privacy
- I am more comfortable at home
- Habit
- Other: _______ *(Please explain)*

24) During the last 2 weeks, how often you have had discomfort due to the following problems

(just one per question, interviewer, please circulate the number)

|  | **Never** | **Sometimes** | **Occationally** |
| --- | --- | --- | --- |
| Little interest or pleasure in doing things | 0 | 1 | 2 |
| Has felt down(a), depressed(a) or hopeless | 0 | 1 | 2 |

**Household exposures**

*In this section, we are interested in learning about your home environment.*

1) How many rooms are in your house? (including bedroom/sleeping areas and excluding bathroom)

| ☐ | ☐ | ☐ | ☐ | ☐ | ☐ | ☐ | ☐ |
| --- | --- | --- | --- | --- | --- | --- | --- |
| 1 | 2 | 3 | 4 | 5 | 6 | 7 | 8 |

2) How many people live in your house, including you (sleep in your house on a typical night, including children)?

| ☐ | ☐ | ☐ | ☐ | ☐ |
| --- | --- | --- | --- | --- |
| 1 | 2-3 | 4-6 | 7-9 | 10+ |

3) How many people in your household earn an income, including yourself?

| ☐ | ☐ | ☐ | ☐ | ☐ |
| --- | --- | --- | --- | --- |
| 0 | 1 | 2 | 3 | 4+ |

4) What type of material are the walls of your house made of? (select all that apply)
   - Baked clay brick
   - Concrete/concrete blocks
   - Mud/adobe/taquezal
   - Stone (quarry)
   - Sheet metal/ Zinc
   - I don't know
   - Other (please explain): _________
5) What materials is your roof made of? *(select all that apply)*
   - Tiles
   - Sheet metal/ Zinc
   - Nicalit
   - Straw or jaragua grass/palm
   - I don't know
   - Other (please explain):_______
6) What is the type of flooring that your house is made of? *(select all that apply)*
   - Dirt floor
   - Concrete/tiling
   - Mud brick
   - Wood
   - Earthenware or ceramics
   - I don't know
   - Other (please explain):

8) Where is the cooking for the household done?

- Inside the house
  - Does the inside cooking area have any of the following? *(Check all that apply)*
    - Window
    - Chimney
    - Exhaust
    - Partially open to outside
    - None
- Outside the house
  - On average how many months per year do you cook outside?

|  |  | Months |
| --- | --- | --- |

9) What is the primary fuel currently used for cooking? (Check only one)

- Propane gas
- Firewood (Wood)
- Charcoal
- Kerosene
- Agricultural waste (corn cobs or dried coffee husks)
- Electricity
- Biogas
- Dried manure
- Dried shrubs/grass
- Other: ___________________ (Specify)

10) For how long you have been using this primary fuel for cooking *(If duration is less than 1 year, please indicate 01)*

|  |  | Years |
| --- | --- | --- |

11) Was there a previous primary fuel used for cooking *(e.g., previously the home used coal, but now uses gas)*?

- No
- Yes

12) If YES to 11: What was the previous primary fuel used for cooking? *(Check only one)*

- Propane gas
- Firewood (Wood)
- Charcoal
- Kerosene
- Agricultural waste (corn cobs or dried coffee husks)
- Electricity
- Biogas
- Dried animal dung
- Dried shrubs/grass
- Other: ___________________ (Specify)

12a) For how long did you use the previous primary fuel for cooking? (If duration is less than 1 year, please indicate 01)

|  |  | Years |
| --- | --- | --- |

**Use of Agrochemicals**

1) Do you use pesticides or herbicides (chemicals to kill weeds, rodents, insects, and/or fungi)?

☐ Yes

☐ No

☐ I don’t know

1a) IF YES to question 1: Do you use pesticides or herbicides at work, as part of your job?

☐ Yes

☐ No

☐ I don’t know

1a1) If YES to question 1a: How often do you use pesticides or herbicides at work?

☐ Every month

☐ Every week

☐ Every day (daily)

☐ Other ____

2) If YES to question 1: Do you use pesticides or herbicides at home, or in other places?

☐ Yes

☐ No

☐ I don’t know

2a) If YES to question 2: How often do you use pesticides or herbicides at your home, on your property, or in places other than your workplace?

☐ Every day (daily)

☐ Every week

☐ Every month

☐ Other _________

3) Which of the following pesticides or herbicides do you use or have you used?

(Interviewer, read the options to the participant and ask if they use the chemical at home or at work.)

☐ I do not use any of these

| Use of chemicals | At Work | At home / On my own property |
| --- | --- | --- |
| Herbicides |  |  |
| Gramoxone or Paraquat Aleman or Aggressive or Superxone or Flame or Rafaga  (active ingredient: paraquat) | ☐ | ☐ |
| Gesapax o Ametrex o Amefor (ingrediente activo: ametrina) | ☐ | ☐ |
| Roundup or Rival or Ranger or Outsider or Warrior or Ranger  (active ingredient: glyphosate) | ☐ | ☐ |
| Acetofor (active ingredient: acetoclor) | ☐ | ☐ |
| Spur or Foram or Pasture (active ingredient: 2,4-D) | ☐ | ☐ |
| Atrazine | ☐ | ☐ |
| Terbutrin | ☐ | ☐ |
| Diuron | ☐ | ☐ |
| Pendimetaline | ☐ | ☐ |
| Other (please specify) | ☐ | ☐ |
| Do not remember (describe, if possible): | ☐ | ☐ |

3b) Insecticides: Which of the following pesticides or herbicides do you use or have you used?

(Interviewer, read the options to the participant and ask if they use the chemical at home or at work.)

☐ I do not use any of these

| Use of chemicals | At Work | At home / On my own property |
| --- | --- | --- |
| Insecticides: |  |  |
| Jade or Joker or Defender (active ingredient: imidacloprides) | ☐ | ☐ |
| Lorsban o Dursban o Forafos o Impacto (ingrediente activo: clorpirifós) | ☐ | ☐ |
| Zipper o Foratox o Foravan o Combate (ingrediente activo: cipermetrina) | ☐ | ☐ |
| Diazide or Dianon or Diazifor (active ingredient: diazinon) | ☐ | ☐ |
| Generic methamide or MTD600 or Monitor (active ingredient: Metamidaphos) | ☐ | ☐ |
| Counter o Terbukill o Terbufos genéricos (ingrediente activo: Terbufoses) | ☐ | ☐ |
| Deltametrina | ☐ | ☐ |
| Permethrin | ☐ | ☐ |
| Parathion or methyl parathion | ☐ | ☐ |
| Endosulfan | ☐ | ☐ |
| Other (please specify) | ☐ | ☐ |
| Do not remember (describe, if possible): | ☐ | ☐ |

3c) Fungicides: Which of the following pesticides or herbicides do you use or have you used?

(Interviewer, read the options to the participant and ask if they use the chemical at home or at work.)

☐ I do not use any of these

| Use of chemicals | At Work | At home / On my own property |
| --- | --- | --- |
| Fungicides: |  |  |
| Mazant (active ingredient: Thiocarbamates) | ☐ | ☐ |
| Foraxil (active ingredient: metalaxyl) | ☐ | ☐ |
| Cupravit (active ingredient: Copper chloride) | ☐ | ☐ |
| Captan | ☐ | ☐ |
| Other (please specify) | ☐ | ☐ |
| Do not remember (describe, if possible): | ☐ | ☐ |

3d) Rodenticides: Which of the following pesticides or herbicides do you use or have you used?

(Interviewer, read the options to the participant and ask if they use the chemical at home or at work)

☐ I do not use any of these

| Use of chemicals | At Work | At home / On my own property |
| --- | --- | --- |
| Rodenticides |  |  |
| Racumin | ☐ | ☐ |
| BioRat | ☐ | ☐ |
| Other (please specify) | ☐ | ☐ |
| Do not remember (describe, if possible): | ☐ | ☐ |

3e) Additional Chemicals: Which of the following pesticides or herbicides do you use or have you used?

(Interviewer, read the options to the participant and ask if they use the chemical at home or at work)

☐ I do not use any of these

| Use of chemicals | At Work | At home / On my own property |
| --- | --- | --- |
| Additional Chemicals |  |  |
| Cyanide | ☐ | ☐ |
| Other (please specify) | ☐ | ☐ |
| Do not remember (describe, if possible): | ☐ | ☐ |

4) Have you applied pesticides or herbicides yourself in the last 48 hours?

☐ Yes

☐ No

☐ I don’t know

5) Did you use personal protective equipment?

☐ Yes

☐ No

☐ Sometimes

**Diet**

*Now I will ask you about your diet.*

1) Does your diet include the following foods? If yes, please specify with what frequency you eat the food product.

| **Type of Food** | **Do you consume this food?** | **If “Yes,” indicate frequency *(Circle “week” or “month”)*** |
| --- | --- | --- |
| Beef | **☐** **Yes**  **☐ No** | **SKIP If NO** |
|  |  | ______/times per week or month |
| Pork | **☐** **Yes**  **☐ No** | **SKIP If NO** |
|  |  | ______/times per week or month |
| Rice | **☐** **Yes**  **☐ No** | **SKIP If NO** |
|  |  | ______/times per week or month |
| Chicken | **☐** **Yes**  **☐ No** | **SKIP If NO**  ______/times per week or month |
| Viscera | **☐** **Yes**  **☐ No** | **SKIP If NO**  ______/times per week or month |
| Seafood / Fish | **☐** **Yes**  **☐ No** | **SKIP If NO**  ______/times per week or month |
| Milk products | **☐** **Yes**  **☐ No** | **SKIP If NO**  ______/times per week or month |
| Arroz | **☐** **Yes**  **☐ No** | **SKIP If NO**  ______/times per week or month |
| Corn or corn products | **☐** **Yes**  **☐ No** | **SKIP If NO**  ______/times per week or month |
| Fresh Fruit | **☐** **Yes**  **☐ No** | **SKIP If NO**  ______/times per week or month |
| Fresh Vegestables | **☐** **Yes**  **☐ No** | **SKIP If NO**  ______/times per week or month |

2) How often do you skip a main meal due to a lack of money?

**☐** Never

**☐** Almost never

**☐** Sometimes

**☐** Very often

**☐** Always

3) In the last year, how often was the following statement true: "The food we had simply ran out (it wasn't enough), and we didn't have money to get more"?

**☐** Never

**☐** Almost never

**☐** Sometimes

**☐** Very often

**☐** Always

**Health- WOMEN only**

1) Generally speaking, do you have your menstrual period?

☐ YES

☐ NO

2) How old were you when you first had your period?

(Interviewer: This is for new participants or those who had not yet started their period at the previous visit.)

3) Are you menstruating today?

☐ YES

☐ NO

☐ I don’t know

**HEALTH - PREGNANCY CARE**

*For WOMEN ONLY:*

1) Have you ever been pregnant?

☐ YES

☐ NO

2) If YES to question one: How old were you when were pregnant for the **first** time? _______
3) If YES to question one: How many children do you have in total? _________
4) What are the ages of your children?

| Biological child | Sex | Age |
| --- | --- | --- |
| Child 1 | ☐M\| ☐F | __________ ☐Months\| ☐ Years |
| Child 2 | ☐M\| ☐F | __________ ☐Months\| ☐ Years |
| Child 3 | ☐M\| ☐F | __________ ☐Months\| ☐ Years |
| Child 4 | ☐M\| ☐F | __________ ☐Months\| ☐ Years |
| Child 5 | ☐M\| ☐F | __________ ☐Months\| ☐ Years |

5) Are you currently pregnant now?

☐ YES

☐ NO

☐ I don’t know

*Ask the following questions to participants who have given birth.*

6) Where did your children’s births take place? Select as many as appropriate for different children.

☐ At home (household/residence)

☐ Hospital

☐ Health Center/Post (primary care clinic)

☐ Maternity Home

☐ Other (specify): ________________________________

7) Have any of your children been born preterm or premature?

☐ Yes

☐ No

☐ I don’t know

7a) IF YES to question 7: Please fill out the table below

| Child | Was your baby born premature? | Sex | Weight – (pounds) |
| --- | --- | --- | --- |
| Child1 | ☐ Yes  ☐ No | ☐M\| ☐F |  |
| Child 2 | ☐ Yes  ☐ No | ☐M\| ☐F |  |
| Child 3 | ☐ Yes  ☐ No | ☐M\| ☐F |  |
| Child 4 | ☐ Yes  ☐ No | ☐M\| ☐F |  |

8) Please describe frequency of visits with a medical professional (doctor, nurse, midwife) and any dietary

supplements or special nutrition you took while pregnant:

Interviewer, this applies only to the participant's current or MOST RECENT pregnancy.

| Trimester | Frequency of medical visits | Supplements | Please describe the given diet |
| --- | --- | --- | --- |
| First trimester: 0-13 weeks of pregnancy |  |  |  |
| Second trimester: 14-26 weeks of pregnancy |  |  |  |
| Third trimester: 27-40 weeks of pregnancy |  |  |  |

9) Did you have or have any of the following medical conditions during pregnancy?

☐ No medical conditions during pregnancy

| Condition | Health problem | Which trimester? | Please describe the treatment completed for this condition in detail ( either at home or through a doctor). Please include the length of treatment |
| --- | --- | --- | --- |
| High risk of miscarriage | ☐Yes \| ☐ No | ☐ \| ☐ \| ☐; 1 \| 2 \| 3 |  |
| Miscarriage | ☐Yes \| ☐ No | ☐ \| ☐ \| ☐; 1 \| 2 \| 3 |  |
| Threatened preterm labor | ☐Yes \| ☐ No | ☐ \| ☐ \| ☐; 1 \| 2 \| 3 |  |
| Hyperemesis gravidarum | ☐Yes \| ☐ No | ☐ \| ☐ \| ☐; 1 \| 2 \| 3 |  |
| Anemia | ☐Yes \| ☐ No | ☐ \| ☐ \| ☐; 1 \| 2 \| 3 |  |
| Urinary tract infection | ☐Yes \| ☐ No | ☐ \| ☐ \| ☐; 1 \| 2 \| 3 |  |
| Sexually transmitted disease | ☐Yes \| ☐ No | ☐ \| ☐ \| ☐; 1 \| 2 \| 3 |  |
| Gestational diabetes | ☐Yes \| ☐ No | ☐ \| ☐ \| ☐; 1 \| 2 \| 3 |  |
| Preeclampsia | ☐Yes \| ☐ No | ☐ \| ☐ \| ☐; 1 \| 2 \| 3 |  |
| Eclampsia | ☐Yes \| ☐ No | ☐ \| ☐ \| ☐; 1 \| 2 \| 3 |  |

10) Did you require bed rest for any medical condition during your pregnancy?

☐ YES

☐ NO

11) Did you breastfeed your child/ren?

☐ YES

☐ NO

11a) ¿Por cuánto tiempo amamantó a sus hijos? (Tiempo en meses): ___________

**Thoughts on the ERC**

*In this section we want to understand your thoughts and concerns regarding chronic kidney disease.*

1) Do you think ERC has affected your community? If **YES**, describe how. _______________________________________________________________________________

**Closing remarks**

We’ve finished our questions for you. Is there anything else you would like to share with us, about your health, your job, or school, your family, or anything else? ____________________________________________________________________________

Interviewer, please write any comments or feedback on the questionnaire applied. Please share any difficulties you have had or perceptions of the participant. Note that your technical response will help the study. ________________________________

**This is the end of the questionnaire. Thank you for participating in our study!**

**To be filled in by the interviewer:**

Time when the interview was completed: _________ (Please enter this into military time)

QA/QC:

2022 ID: __________________

Notes: ______________________
